# Smaller Ischiofemoral Space Is Associated With Proximal Hamstring Injuries

**DOI:** 10.1002/ars2.70141

**Published:** 2026-10-31

**Authors:** Thun Itthipanichpong, Julia Retzky, Patrick Curran, Samarth V. Menta, Anil S. Ranawat

**Affiliations:** ^1^ The Hospital for Special Surgery New York New York U.S.A.; ^2^ Department of Orthopaedics Chulalongkorn University and King Chulalongkorn Memorial Hospital Bangkok Thailand; ^3^ University of Connecticut School of Medicine Farmington Connecticut U.S.A.

## Abstract

**Purpose:**

To develop a reference ischiofemoral space (IFS) value for patients with proximal hamstring injuries (HSI) and to compare it with individuals without proximal hamstring pathology.

**Methods:**

We retrospectively collected the data from our institution. Patients aged 18 years or older with a proximal HSI were included. Patients with insufficient imaging data, those with prior hip surgery, those with concurrent femoroacetabular impingement syndrome, and those with concurrent ischiofemoral impingement as defined by quadratus femoris edema on magnetic resonance imaging (MRI) were excluded. A 1:2 matched‐cohort analysis was conducted. Two cohorts were (1) patients with proximal HSI and (2) patients with femoroacetabular impingement syndrome as a control cohort (Control). Both pelvic anteroposterior (AP) radiographs and axial MRI were used to assess IFS and were compared between the 2 cohorts. Additionally, the neck‐shaft angle was collected and compared.

**Results:**

There were 60 patients in the HSI cohort and 124 patients in the control cohort. The mean neck‐shaft angle, IFS from the AP pelvis, and IFS from the axial MRI for the HSI cohort were 136.35° ± 5.63°, 24.35 ± 8.60 mm, and 26.57 ± 7.96 mm, respectively. For the control cohort, these values were 135.30° ± 4.53°, 29.83 ± 9.84 mm, and 29.28 ± 8.54 mm, respectively. The IFS in the HSI group was significantly lower than that in the control group on both pelvic AP radiograph (*P* < .001) and axial MRI (*P* = .036).

**Conclusions:**

Patients with proximal HSI had a smaller IFS compared with those with femoroacetabular impingement syndrome.

**Level of Evidence:**

Level III, retrospective cohort study.

## Graphical abstract

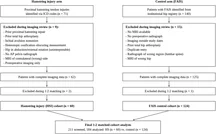

Hamstring injuries (HSI) are a prevalent type of muscle injury, accounting for 25% to 30% of all muscle injuries and the most common in the body.^1^ The hamstring muscle complex comprises 3 muscles, the biceps femoris (long and short heads), semitendinosus, and semimembranosus, which originate from the ischial tuberosity and span both the hip and knee joints.^2^ This biarticular configuration renders the hamstrings particularly susceptible to injury during activities requiring simultaneous hip flexion and knee extension, such as sprinting, hurdling, and kicking.^3^ Among these injuries, up to 10% involve a tear or avulsion at the proximal hamstring origin.^4^ Proximal HSI encompass a spectrum from chronic tendinopathy to partial tears and complete avulsions, and their management varies accordingly; myotendinous strains are generally treated conservatively, whereas complete avulsions often require surgical repair.^1^, ^4^

The ischiofemoral space (IFS) is a potential space located between the ischial tuberosity and the lesser trochanter (LT) of the femur.^5^ The quadratus femoris muscle lies in the IFS.^6^ Restricted IFS has been identified as a potential risk factor for proximal hamstring ruptures because of the close physical proximity of the LT and the ischium.^7^, ^8^, ^9^ Several anatomic factors may contribute to a reduced IFS, including coxa valga, increased or decreased femoral version, and pelvic morphologic variations.^10^, ^11^ Ischiofemoral impingement (IFI) represents the extreme of IFS narrowing, resulting in compression of the quadratus femoris muscle and associated edema. IFI may present with pain because of compression of the quadratus femoris muscle, with secondary involvement of the sciatic nerve and proximal hamstring tendons.^12^ However, subclinical reductions in IFS that do not meet criteria for IFI may still predispose to proximal hamstring pathology.^13^, ^14^

Importantly, the clinical presentation of proximal HSI overlaps considerably with several other posterior hip and deep gluteal pathologies, creating significant diagnostic challenges. Deep gluteal syndrome is an increasingly recognized entity characterized by sciatic nerve entrapment in the deep gluteal space, which may arise from compression by the piriformis muscle, abnormalities of the gemelli‐obturator internus complex, or fibrous bands.^15^, ^16^ Hamstring syndrome, first described by Puranen and Orava, refers to chronic gluteal sciatic pain caused by fibrotic adhesions that tether the sciatic nerve to the proximal hamstring origin at the ischial tuberosity, and it is particularly common in endurance athletes.^17^ Given that the sciatic nerve passes approximately 1.2 cm lateral to the ischial tuberosity, proximal hamstring tendon pathology, including tendinopathy, partial tears, and scar tissue from prior injuries, can readily cause irritation or entrapment of the nerve.^7^, ^18^ Additionally, sacroiliac joint dysfunction and lumbar‐referred pain may mimic or coexist with proximal hamstring pathology, further complicating the diagnostic picture.^19^ These overlapping conditions point to the need for a thorough understanding of posterior hip anatomy and a systematic approach to differential diagnosis when evaluating patients with posterior hip and buttock pain.

Sciatic nerve entrapment, IFI, and tears of the proximal origin of the hamstring are among the recognized subgluteal space pathologies.^7^, ^20^ Although these conditions are individually well described, the relation between IFS dimensions and the risk of proximal HSI remains poorly understood. Torriani et al. showed that 6 out of 12 hips in their study exhibited proximal hamstring pathologies associated with IFI.^13^ Martin et al. noted that IFI and hamstring syndrome can coexist and the evaluation of posterior hip pain requires assessment at the osseous, capsulolabral, musculotendinous, neurovascular, and kinematic levels.^9^ Despite these observations, there is a paucity of literature specifically examining whether subclinical IFS narrowing, below the threshold for IFI diagnosis, constitutes an independent anatomical risk factor for proximal HSI.

The purposes of this study were to develop a reference IFS value for patients with proximal HSI and to compare it with that of individuals without proximal hamstring pathology. We hypothesized that patients with proximal HSI will have a significantly smaller IFS compared with individuals without such injuries.

## METHODS

After Institutional Review Board approval, a retrospective cohort study was conducted using data from the hip registry and radiographic records from the Hospital for Special Surgery. Patients with complete proximal hamstring tears were 1:2 age‐sex‐body mass index matched to patients with femoroacetabular impingement syndrome (FAIS) only, which served as controls.

Patients aged 18 years or older with partial or complete proximal hamstring tendon injuries were initially identified using relevant International Classification of Diseases diagnosis codes. The diagnosis of proximal HSI was then confirmed through review of clinical documentation revealing signs and symptoms consistent with hip/proximal hamstring involvement, along with magnetic resonance imaging (MRI) findings confirming a tear of the proximal hamstring tendons. Patients were excluded if they had injuries involving the myotendinous junction, isolated proximal hamstring tendinosis, hip osteoarthritis, prior hip surgery, concomitant FAIS, a known diagnosis of IFI, or concomitant edema of the quadratus femoris muscle suggestive of coexisting IFI.^21^

The control group consisted of patients diagnosed with FAIS. Diagnostic criteria for FAIS included alpha angle greater than 60° at the anterosuperior head‐neck junction or an anterior femoral offset of less than 8 mm.^22^ We selected FAIS patients as the control group because of the availability of a large database and the distinct anterior pathology of FAIS, contrasting with the posterior pathology associated with hamstring and IFI. Therefore, the likelihood of concomitant pathology was considered low.

### Data Collection

Demographic data, including age, sex, body mass index, and laterality, were collected for all participants. Imaging data related to IFS were obtained for both the HSI and the Control groups. These imaging measurements were taken from T2‐weighted axial MRI and anteroposterior (AP) pelvis radiograph images. Standing AP pelvis radiographs were obtained per our institution's standard protocol, with feet internally rotated approximately 15° to neutralize femoral anteversion. MRI was performed in the supine position on a 1.5 or 3 T scanner using a standard hip protocol, including axial T2‐weighted sequences. Neck‐shaft angle (NSA) was measured on AP pelvis radiographs only.

The IFS was measured as the narrowest distance between the LT and the ischium on both AP pelvis radiograph (Figure 1) and T2‐weighted axial MRI scans^23^ (Figure 2). The NSA was measured on standard AP pelvis radiographs as the angle between the femoral neck axis and the femoral shaft axis (Figure 3).

**FIGURE 1:**
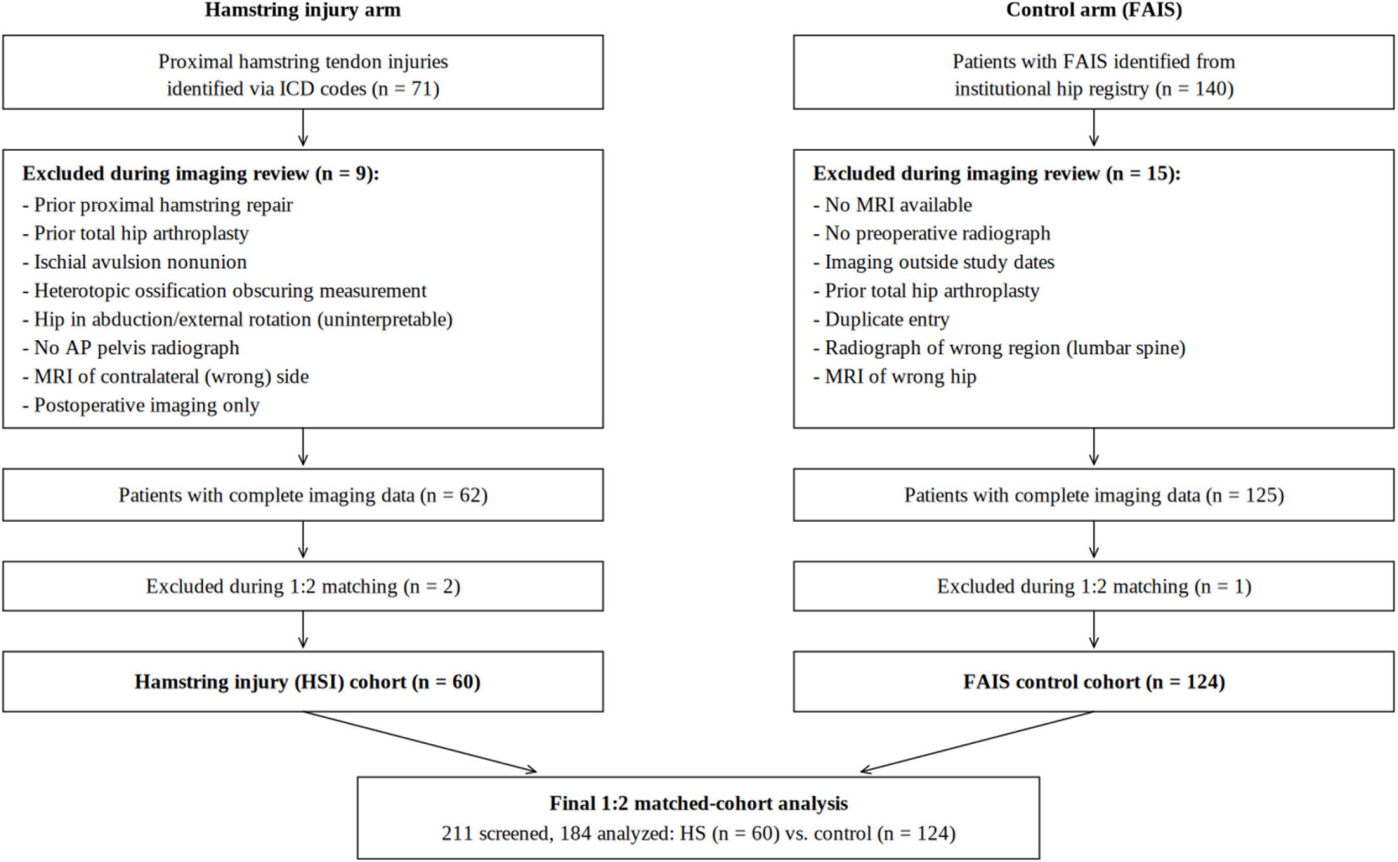
Flowchart for patients included in the study. (AP, anteroposterior; FAIS, femoroacetabular impingement syndrome; ICD, International Classification of Diseases; MRI, magnetic resonance imaging.)

**FIGURE 2:**
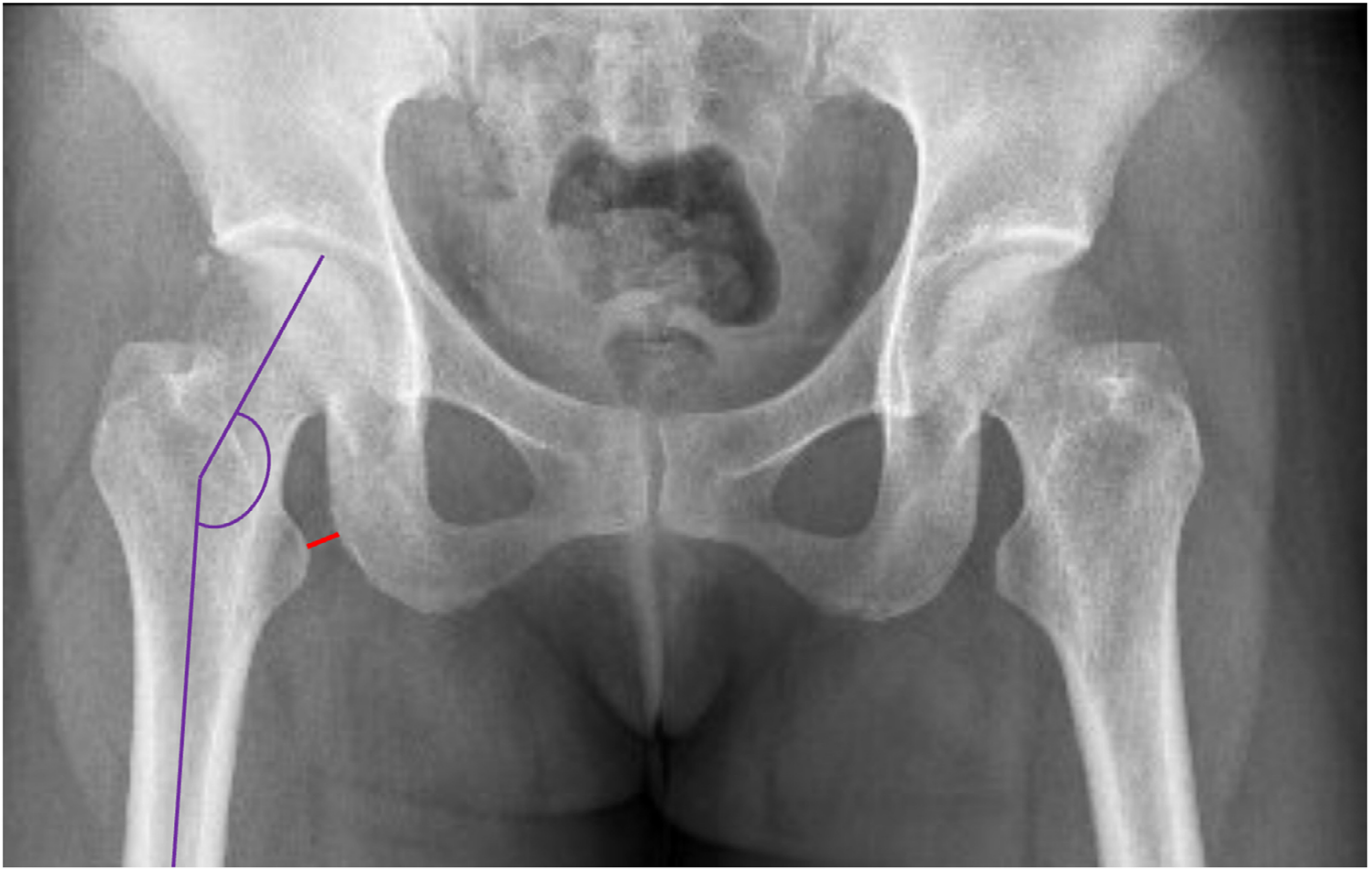
A radiograph of both hips (AP view) shows the method of measuring the NSA (purple line), which is the angle connecting the axis of the femoral neck to the axis of the femoral shaft. The IFS was also measured from the pelvis AP radiograph (red line), representing the narrowest distance between the lesser trochanter and the ischium. (AP, anteroposterior; IFS, Ischiofemoral space; NSA, neck‐shaft angle.)

**FIGURE 3:**
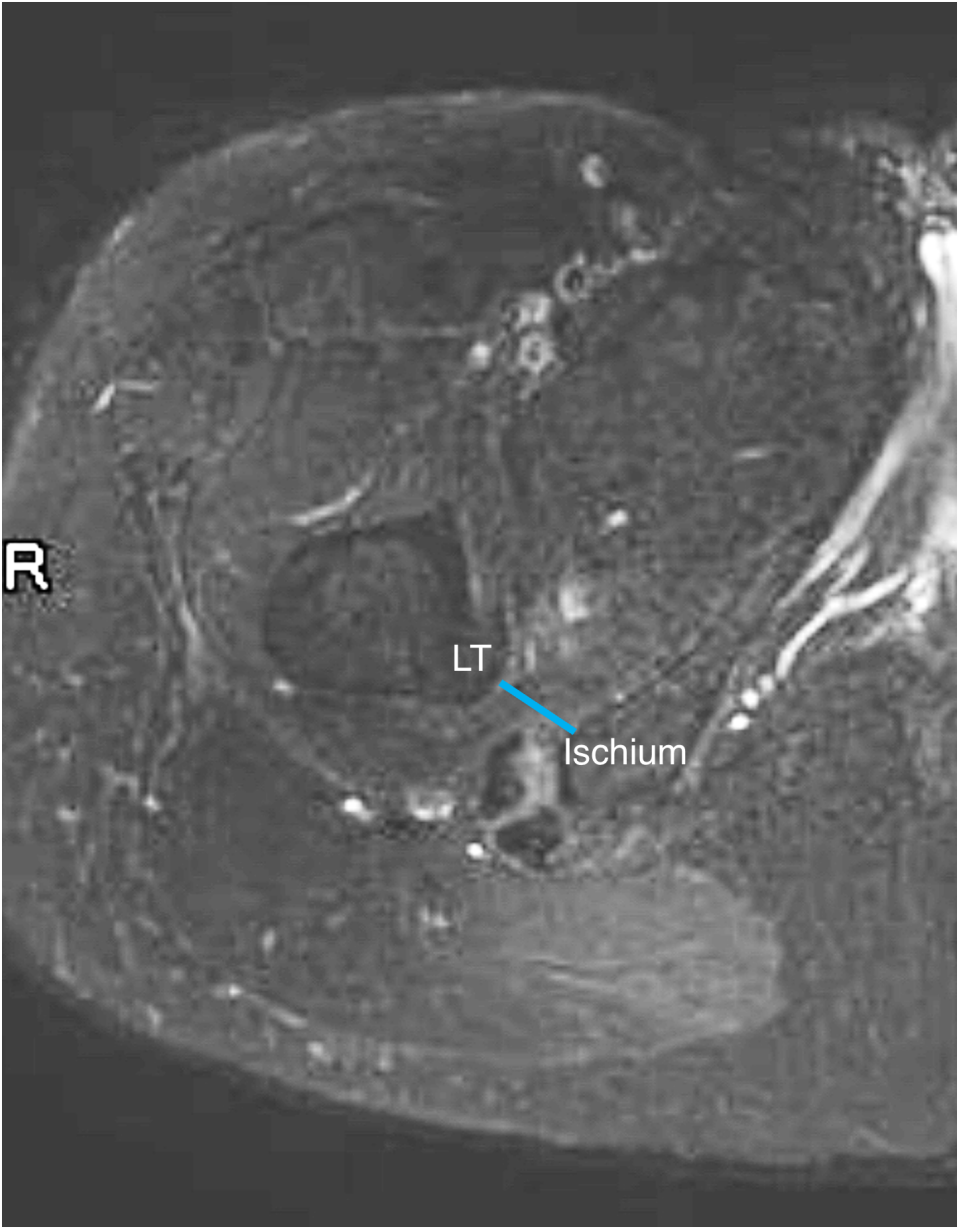
The axial MRI T2 of the right hip shows the method of IFS measurement from the axial view (blue line). The measurement was taken at the narrowest space between the LT and the ischium. (IFS, Ischiofemoral space; LT, Lesser trochanter; MRI, Magnetic resonance imaging.)

Each measurement was performed independently by 2 authors of this study and repeated twice, with at least a 2‐week interval between measurements, to assess inter‐ and intraobserver reliabilities. The 2 authors who performed the measurements were an orthopaedic surgery resident (now a sports medicine fellow, J.S.R.) and the other a fellowship‐trained sports medicine attending physician (T.I.). Intraclass correlation coefficients (ICCs) for intraobserver and interobserver reliabilities were calculated using a 2‐way random effects model for absolute agreement. ICC values were interpreted per Cicchetti's (1994) guidelines: <0.40 poor, 0.40 to 0.59 fair, 0.60 to 0.74 good, and 0.75 to 1.00 excellent.^24^

### Data Analysis

Descriptive statistics, including means and standard deviations, were provided for demographic data. Mean comparisons for each variable were conducted using paired *t* tests. To ensure accuracy, 2 separate investigators measured each parameter on 2 separate occasions, and the average values were used for analysis. Statistical significance was defined as *P* < .05.

## RESULTS

Sixty patients were included in the HSI cohort (20 men and 40 women), and 124 age‐ and sex‐matched patients were included in the FAIS group (50 men and 74 women). Chart review of operative notes and radiology reports was performed to characterize the injury composition of the HSI cohort. Most injuries were traumatic in mechanism (56/60, 93.3%), with the remainder being atraumatic or degenerative (4/60, 6.7%). Regarding tear severity, 36 (60.0%) were complete or near‐complete tears, 16 (26.7%) were avulsions, and 8 (13.3%) were partial‐thickness tears. The mean age was 50.35 ± 12.49 years in the HSI cohort and 43.91 ± 7.74 years in the Control cohort. There was a statistically significant difference in age between the 2 groups (*P* < .001). The sex distribution did not differ significantly between cohorts (*P* = .360).

Both radiograph and MRI measurements revealed good to excellent intraobserver reliability. The ICCs for the NSA, IFS from radiograph, and IFS from MRI were 0.74, 0.97, and 0.78, respectively, for reviewer 1, and 0.93, 0.94, and 0.99, respectively, for reviewer 2. The interobserver agreement for the NSA, IFS from radiograph, and IFS from MRI was 0.91, 0.63, and 0.66, respectively.

The mean NSA measured 136.35° ± 5.63° in the HSI group and 135.30° ± 4.53° in the control group. There was no statistically significant difference between the groups (*P* = .207), with a mean difference of 1.06°. The IFS measurements from the AP pelvis radiograph and axial MRI were 24.35 ± 8.60 and 26.57 ± 7.96 mm, respectively, in the HSI group, whereas, in the control group, the IFS measurements were 29.83 ± 9.84and 29.28 ± 8.54 mm, respectively. Comparing these 2 cohorts, the IFS in the HSI group was significantly smaller than that of the control group in both the pelvic AP radiograph (*P* < .001) and the axial MRI (*P* = .036; Table 1).

**TABLE 1:** The Measurement Data Compared Between the Hamstring Injury Patients and the Control Group

	**Hamstring Group (HSI)**	**Control Cohort (Control)**	***P*** **Value**
	**Mean ± SD (Range)**	**Mean ± SD (Range)**	
NSA	136.35° ± 5.63°	135.30° ± 4.53°	.207
IFS from pelvis AP radiograph	24.35 ± 8.60 mm	29.83 ± 9.84 mm	<.001∗
IFS from axial MRI	26.57 ± 7.96 mm	29.28 ± 8.54 mm	.036∗

AP, anteroposterior; HSI, hamstring injury; IFS, ischiofemoral space; MRI, magnetic resonance imaging; NSA, neck‐shaft angle; SD, standard deviation.

∗ Statistical significance at *P* < .05.

In sex‐disaggregated analyses (Table 2), male patients in the HSI cohort (n = 20) had significantly smaller IFS measurements compared with male controls (n = 50) on both AP pelvis radiographs (28.46 ± 9.50 mm vs 35.28 ± 9.97 mm, *P* = .011) and axial MRI (28.77 ± 6.67 mm vs 32.95 ± 8.02 mm, *P* = .031). There was no significant difference in NSA between male subgroups (135.20° ± 4.84° vs 135.38° ± 4.91°, *P* = .893). Among women, the HSI cohort (n = 40) had significantly smaller IFS on AP pelvis radiographs compared with female controls (n = 74) (22.30 ± 7.42 mm vs 26.15 ± 7.90 mm, *P* = .012), but the difference on axial MRI did not reach statistical significance (25.46 ± 8.39 mm vs 26.80 ± 8.01 mm, *P* = .412). The NSA did not differ significantly between female subgroups (136.93° ± 5.96° vs 135.24° ± 4.29°, *P* = .118).

**TABLE 2:** Sex‐Disaggregated Analysis of Measurements Between Hamstring Injury Patients and the Control Group

	**HSI Group**	**Control Group**	***P*** **Value**	**Cohen's** ***d***
Men (HSI n = 20, Control n = 50)				
NSA	135.20° ± 4.84°	135.38° ± 4.91°	.893	‐
IFS from pelvis AP radiograph	28.46 ± 9.50 mm	35.28 ± 9.97 mm	.011∗	–0.69 (medium)
IFS from axial MRI	28.77 ± 6.67 mm	32.95 ± 8.02 mm	.031∗	–0.55 (medium)
Women (HSI n = 40, Control n = 74)				
NSA	136.93° ± 5.96°	135.24° ± 4.29°	.118	‐
IFS from pelvis AP radiograph	22.30 ± 7.42 mm	26.15 ± 7.90 mm	.012∗	–0.50 (medium)
IFS from axial MRI	25.46 ± 8.39 mm	26.80 ± 8.01 mm	.412	‐

AP, anteroposterior; HSI, hamstring injury; IFS, ischiofemoral space; MRI, magnetic resonance imaging; NSA, neck‐shaft angle.

∗ Statistical significance at *P* < .05.

## DISCUSSION

The most important finding of this study is that the IFS, as measured on both pelvis AP radiographs and axial MRI scans, was consistently smaller in individuals with proximal HSI compared with those without proximal HSI. Reduced IFS may predispose patients to proximal HSI.

The IFS was measured using both AP pelvis radiographs and axial MRI, with mean values of 24.35 ± 8.60 and 26.57 ± 7.96 mm, respectively, in the HSI cohort. These values exceed conventional IFI thresholds (Torriani et al.: 13 ± 5 mm; Singer et al.: 14.9 ± 4.8 mm).^13^, ^14^ This finding indicates that the association between reduced IFS and proximal HSI exists along a continuum, rather than being confined to patients who meet established IFI diagnostic criteria. Torriani et al. first identified this relation, reporting that 50% of hips with IFI had hamstring tendon edema and 25% had partial hamstring tears.^13^ Tay et al. subsequently described a case in which IFI masked concurrent proximal hamstring tendinopathy, illustrating the clinical overlap between these conditions.^8^ Martin et al. further emphasized that IFI and hamstring syndrome can coexist. That evaluation of posterior hip pain requires systematic assessment at the osseous, capsulolabral, musculotendinous, neurovascular, and kinematic levels.^9^ More recently, Heimann et al. examined 256 hips in young patients undergoing evaluation for joint‐preserving hip surgery and found a 9% prevalence of IFI, with a significantly higher prevalence of 16% in women.^10^ Their multivariate analysis identified female sex, high femoral torsion (excessive anteversion), and an ischial angle greater than 127° as independent imaging predictors of IFI, reinforcing that multiple osseous parameters beyond the IFS measurement contribute to the pathomechanics of ischiofemoral narrowing.^10^ The present findings extend this body of literature by revealing that even subclinical reductions in IFS, well above established IFI thresholds, are associated with proximal HSI.

The clinical significance of these findings should be considered within the broader context of posterior hip and deep gluteal pathology. Proximal HSI share overlapping clinical features with several conditions, including deep gluteal syndrome, hamstring syndrome, sciatic nerve entrapment, IFI, and sacroiliac joint dysfunction.^9^, ^16^, ^19^ The close anatomical relation between the proximal hamstring origin, the quadratus femoris muscle, and the sciatic nerve within the deep gluteal space allows pathology in one structure to readily affect adjacent structures.^25^ For instance, a reduced IFS may predispose to mechanical irritation of the proximal hamstring tendon and may also contribute to compression of the quadratus femoris and secondary irritation of the sciatic nerve, thereby blurring the distinction between IFI, hamstring syndrome, and deep gluteal syndrome.^7^, ^9^, ^15^ This diagnostic overlap brings attention to the importance of comprehensive imaging evaluation, including MRI assessment of the IFS, quadratus femoris, and sciatic nerve, when evaluating patients with posterior hip and buttock pain.^12^, ^25^

The relation between femoral NSA and IFS dimensions has been investigated in the IFI literature, yielding conflicting results. Tosun et al. reported a significantly greater inclination angle in patients with quadratus femoris edema compared with controls, and Bredella et al. similarly found that patients with IFI have increased femoral neck angles, suggesting that coxa valga may be associated with ischiofemoral narrowing.^11^, ^26^ Stenhouse et al. confirmed this association in the pediatric population, showing significantly larger inclination angles in children with IFI (145.5° vs 138.7°, *P* <.05).^27^ However, Gardner et al. examined 100 consecutive hips with hip pain. They did not find a statistically significant difference in NSA between patients with and without quadratus femoris edema, concluding that IFI may be a dynamic syndrome not solely explained by static bony parameters.^28^ In the present study, the mean NSA was numerically higher in the HSI cohort (136.35° vs 135.30°); however, this difference did not reach statistical significance (*P* = .207), with a mean difference of only 1.06°. This finding aligns with the growing recognition that IFS narrowing is multifactorial, involving the interplay of femoral version, pelvic morphology, ischial angle, and dynamic factors such as gait pattern and hip rotation, rather than being attributable to any single osseous parameter.^10^, ^11^, ^12^

In addition to the NSA, several other bony anatomical factors may influence the IFS and contribute to the risk of proximal HSI. Femoral version, particularly increased anteversion or retroversion, has been implicated in altering the spatial relation between the LT and the ischium.^29^, ^30^ Lerch et al. showed that patients with increased femoral version can develop posterior extra‐articular IFI involving both the lesser and greater trochanters.^30^ Pelvic morphology, including the position and orientation of the ischial tuberosities, may also play a role. Women tend to have wider pelvic outlets with more everted ischial tuberosities, which may partly explain the higher prevalence of IFI in women.^11^, ^14^ Future studies incorporating 3‐dimensional computed tomography analysis of femoral version, pelvic morphology, and IFS may better characterize these complex anatomical relations and their contribution to proximal HSI risk.^12^, ^31^

Sex‐disaggregated analyses showed that the association between reduced IFS and proximal HSI was present in both sexes on AP pelvis radiographs, with significant differences observed in men (*P* = .011, Cohen's *d* = –0.69) and women (*P* = .012, Cohen's *d* = –0.50). On axial MRI, however, the difference reached significance only in men (*P* = .031, Cohen's *d* = –0.55) and not in women (*P* = .412). The larger effect sizes observed in men may reflect sex‐based differences in pelvic morphology; women generally have a wider pelvic outlet and more laterally positioned ischial tuberosities, which may attenuate the IFS narrowing associated with proximal hamstring pathology.^11^, ^14^ Additionally, the smaller IFS values observed in the female HSI cohort on both modalities (22 mm on radiograph and 25.46 mm on MRI) compared with male patients (28.46 and 28.77 mm, respectively) suggest that baseline sex‐related differences in IFS dimensions may influence the threshold at which IFS narrowing becomes clinically relevant. The NSA did not differ significantly between the HSI and control subgroups in either sex (men, *P* = .893; women, *P* = .118), further supporting the observation that the NSA alone does not explain the IFS differences observed between groups. These findings highlight the importance of sex‐specific normative data when evaluating IFS measurements and indicate the need for further investigation in larger cohorts to characterize better sex‐based differences in the relation between IFS and proximal HSI risk.^31^, ^32^

### Limitations

Several limitations should be considered in interpreting these results. First, the retrospective study design inherently limits the ability to control for confounding variables and to establish causality between reduced IFS and proximal HSI. Second, no a priori power analysis was performed to determine the minimum sample size required to detect clinically meaningful differences in IFS between groups, which may result in the study being underpowered to detect smaller but clinically relevant effect sizes, particularly in subgroup analyses. Third, the study was conducted at a single tertiary referral center, which may limit the generalizability of the findings to other clinical settings and patient populations.

The control group consisted of patients with FAIS, which may not fully represent the general population. FAIS patients exhibit distinct hip morphology, including cam and/or pincer deformities, which could independently influence bony anatomy around the hip and the IFS. Additionally, the selective exclusion of concurrent FAIS from the HSI cohort, while using FAIS as the control group, introduces an inherent asymmetry in group composition that may limit comparability between the 2 cohorts. Furthermore, despite the use of a 1:2 age‐sex‐body mass index matching protocol, a significant age discrepancy persisted between the HSI cohort (mean 50.35 ± 12.49 years) and the FAIS control cohort (mean 43.91 ± 7.74 years). This represents a notable limitation, as age‐related changes in pelvic and femoral morphology, bone density, and soft tissue quality may confound IFS measurements and the association with proximal HSI.

Several potentially important morphologic and imaging parameters were not assessed. Data on femoral version, a known contributor to IFS narrowing and posterior extra‐articular IFI, were not collected.^29^, ^30^ Similarly, other morphologic variables that may influence the IFS, such as pelvic incidence, ischial tuberosity morphology, and acetabular version, were not evaluated. Range‐of‐motion data were also not collected, despite the relevance of hip positioning, particularly rotation and extension, in dynamically altering the IFS.^33^ Additionally, imaging was limited to AP pelvis radiographs and axial MRI. A false profile (Lequesne) view of the pelvis, which provides a lateral perspective that may better characterize the spatial relation between the ischium and the LT, was not obtained. Furthermore, cross‐sectional computed tomography, including 3‐dimensional reconstructions, was not used. Computed tomography is increasingly regarded as the reference standard for quantifying bony morphologic parameters of the hip, including femoral version, ischial morphology, and the 3‐dimensional geometry of the IFS, and offers superior accuracy compared with 2‐dimensional radiographic measurements.^10^, ^30^ The reliance on AP radiographs and axial MRI in the present study may therefore underestimate the true complexity of the osseous relations contributing to IFS narrowing.

IFS measurements are inherently dependent on patient positioning at the time of imaging. Itthipanichpong et al. showed that hip external rotation significantly decreases the ischiofemoral distance, and variations in hip rotation, abduction/adduction, and flexion/extension during imaging acquisition can meaningfully alter the measured IFS.^33^ In this study, the quality and standardization of pelvic AP radiographs were difficult to control retrospectively. Rotational variability of the pelvis and femur at the time of radiograph acquisition introduces measurement error, as even small degrees of pelvic tilt or femoral rotation can change the projected distance between the LT and the ischium. Although 2 independent reviewers performed multiple measurements and the results were averaged, the inherent positioning dependence of IFS on plain radiographs remains a limitation.

Chart review of operative notes revealed that the HSI cohort was predominantly traumatic (93%), with most being complete or near‐complete tears (60%) or avulsions (27%), and relatively few partial‐thickness tears (13%). Subgroup analyses based on injury severity (e.g., partial vs complete tears, the number of tendons involved, or degree of tendon retraction) or mechanism (acute traumatic vs chronic degenerative) were not performed. The heavily skewed distribution of injury types within the cohort precluded meaningful statistical comparison between these subgroups. It is plausible that IFS measurements may differ across injury severity grades, with more severe injuries potentially being associated with smaller IFS values. Similarly, analyses were not stratified by specific tendon involvement (biceps femoris, semitendinosus, or semimembranosus) or chronicity of injury.

## CONCLUSIONS

Patients with proximal HSI had a smaller IFS compared with those with FAIS.

## DISCLOSURES

The author (A.S.R.) declares the following financial interests/personal relationships which may be considered as potential competing interests: A.S.R. reports grants or contracts from the Smith & Nephew, Bodycad, Cervos Medical, Overture, Native, and Moximed (researcher/research support relationship); consulting fees from Smith & Nephew, Bodycad, Cervos Medical, and Overture (paid consultant); stock options from Overture and Bodycad; and serves as a board member for Bodycad. The other authors (T.I., J.R., P.C., S.V.M.) declare that they have no known competing financial interests or personal relationships that could have appeared to influence the work reported in this article.

## References

[1] Arner JW , McClincy MP , Bradley JP . Hamstring injuries in athletes: Evidence‐based treatment. J Am Acad Orthop Surg. 2019;27:868‐877. 10.5435/JAAOS-D-18-00741 31283532

[2] Beltran L , Ghazikhanian V , Padron M , Beltran J . The proximal hamstring muscle‐tendon‐bone unit: A review of the normal anatomy, biomechanics, and pathophysiology. Eur J Radiol. 2012;81:3772‐3779. 10.1016/j.ejrad.2011.03.099 21524864

[3] Lempainen L , Johansson K , Banke IJ , et al. Expert opinion: Diagnosis and treatment of proximal hamstring tendinopathy. Muscles Ligaments Tendons J. 2015;5:23‐28. PMC4396672 25878983

[4] van der Made AD , Reurink G , Gouttebarge V , Tol JL , Kerkhoffs GM . Outcome after surgical repair of proximal hamstring avulsions: A systematic review. Am J Sports Med. 2015;43:2841‐2851. 10.1177/0363546514555327 25384502

[5] Kivlan BR , Martin RL , Martin HD . Ischiofemoral impingement: Defining the lesser trochanter‐ischial space. Knee Surg Sports Traumatol Arthrosc. 2017;25:72‐76. 10.1007/s00167-016-4036-y 26869034

[6] Gollwitzer H , Banke IJ , Schauwecker J , Gerdesmeyer L , Suren C . How to address ischiofemoral impingement? Treatment algorithm and review of the literature. J Hip Preserv Surg. 2017;4:289‐298. 10.1093/jhps/hnx035 PMC5721376 29250337

[7] Maldonado DR . Editorial commentary: Subgluteal hip arthroscopy for sciatic nerve entrapment, ischiofemoral impingement, or proximal hamstring tears: Beyond the comfort zone. Arthroscopy. 2021;37:2162‐2163. 10.1016/j.arthro.2021.03.054 34226006

[8] Tay MRJ , Farag J . Ischiofemoral impingement syndrome masked by proximal hamstring tendinopathy as a cause of gluteal pain in a marathon runner. Am J Phys Med Rehabil. 2024;103:e77‐e79. 10.1097/PHM.0000000000002404 38206644

[9] Martin HD , Khoury A , Schröder R , Palmer IJ . Ischiofemoral impingement and hamstring syndrome as causes of posterior hip pain: Where do we go next? Clin Sports Med. 2016;35:469‐486. 10.1016/j.csm.2016.02.010 27343397

[10] Heimann AF , Wagner M , Vavron P , et al. Ischiofemoral impingement in joint preserving hip surgery: Prevalence and imaging predictors. Insights Imaging. 2025;16:78. 10.1186/s13244-025-01946-2 PMC11971088 40186048

[11] Bredella MA , Azevedo DC , Oliveira AL , et al. Pelvic morphology in ischiofemoral impingement. Skeletal Radiol. 2015;44:249‐253. 10.1007/s00256-014-2041-0 25371087

[12] Torriani M . Ischiofemoral impingement syndrome in 2024: Updated concepts and imaging methods. Magn Reson Imaging Clin N Am. 2025;33:63‐73. 10.1016/j.mric.2024.06.005 39515961

[13] Torriani M , Souto SCL , Thomas BJ , Ouellette H , Bredella MA . Ischiofemoral impingement syndrome: An entity with hip pain and abnormalities of the quadratus femoris muscle. AJR Am J Roentgenol. 2009;193:186‐190. 10.2214/AJR.08.2090 19542413

[14] Singer AD , Subhawong TK , Jose J , Tresley J , Clifford PD . Ischiofemoral impingement syndrome: A meta‐analysis. Skeletal Radiol. 2015;44:831‐837. 10.1007/s00256-015-2111-y 25672947

[15] Martin HD , Shears SA , Johnson JC , Smathers AM , Palmer IJ . The endoscopic treatment of sciatic nerve entrapment/deep gluteal syndrome. Arthroscopy. 2011;27:172‐181. 10.1016/j.arthro.2010.07.008 21071168

[16] Park JW , Lee YK , Lee YJ , Shin S , Kang Y , Koo KH . Deep gluteal syndrome as a cause of posterior hip pain and sciatica‐like pain. Bone Joint J. 2020;102‐B:556‐567. 10.1302/0301-620X.102B5.BJJ-2019-1212.R1 32349600

[17] Puranen J , Orava S . The hamstring syndrome. A new diagnosis of gluteal sciatic pain. Am J Sports Med. 1988;16:517‐521. 10.1177/036354658801600515 3189686

[18] Saikku K , Vasenius J , Saar P . Entrapment of the proximal sciatic nerve by the hamstring tendons. Acta Orthop Belg. 2010;76:321‐324. 20698451

[19] Goom TSH , Malliaras P , Reiman MP , Purdam CR . Proximal hamstring tendinopathy: Clinical aspects of assessment and management. J Orthop Sports Phys Ther. 2016;46:483‐493. 10.2519/jospt.2016.5986 27084841

[20] Leite MJ , Pinho AR , Silva MR , Lixa JC , Madeira MD , Pereira PG . Deep gluteal space anatomy and its relationship with deep gluteal pain syndromes. HIP Int. 2022;32:510‐515. 10.1177/1120700020966255 33043696

[21] Griffin DR , Dickenson EJ , O'Donnell J , et al. The Warwick agreement on femoroacetabular impingement syndrome (FAI syndrome): An international consensus statement. Br J Sports Med. 2016;50:1169‐1176. 10.1136/bjsports-2016-096743 27629403

[22] Albers CE , Wambeek N , Hanke MS , Schmaranzer F , Prosser GH , Yates PJ . Imaging of femoroacetabular impingement: Current concepts. J Hip Preserv Surg. 2016;3:245‐261. 10.1093/jhps/hnw035 PMC5883171 29632685

[23] Greenky M , Cohen SB . Magnetic resonance imaging for assessing hamstring injuries: Clinical benefits and pitfalls – A review of the current literature. Open Access J Sports Med. 2017;8:167‐170. 10.2147/OAJSM.S113007 PMC5522675 28761382

[24] Cicchetti DV . Guidelines, criteria, and rules of thumb for evaluating normed and standardized assessment instruments in psychology. Psychol Assess. 1994;6:284‐290.

[25] Hanna AS , Schmidt BT , Kanarek AA , et al. Anatomical proximity between sciatic nerve and ischial spine and its relationship to the development of deep gluteal pain syndrome. World Neurosurg. 2024;188:e367‐e375. 10.1016/j.wneu.2024.05.117 38796142

[26] Tosun O , Algin O , Yalcin N , Cay N , Ocakoglu G , Karaoglanoglu M . Ischiofemoral impingement: Evaluation with new MRI parameters and assessment of their reliability. Skeletal Radiol. 2012;41:575‐587. 10.1007/s00256-011-1257-5 21874607

[27] Stenhouse G , Kaiser S , Kelley SP , Stimec J . Ischiofemoral impingement in children: Imaging with clinical correlation. AJR Am J Roentgenol. 2016;206:426‐430. 10.2214/AJR.15.14909 26587798

[28] Gardner SS , Dong D , Peterson LE , Park KJ , Harris JD . Is there a relationship between femoral neck‐shaft angle and ischiofemoral impingement in patients with hip pain? J Hip Preserv Surg. 2020;7:43‐48. 10.1093/jhps/hnaa006 PMC7195935 32382428

[29] Khoury AN , Hatem M , Bowler J , Martin HD . Hip–spine syndrome: Rationale for ischiofemoral impingement, femoroacetabular impingement and abnormal femoral torsion leading to low back pain. J Hip Preserv Surg. 2021;7:390‐400. 10.1093/jhps/hnaa054 PMC8081421 33948195

[30] Lerch TD , Zwingelstein S , Schmaranzer F , et al. Posterior extra‐articular ischiofemoral impingement can be caused by the lesser and greater trochanter in patients with increased femoral version: Dynamic 3D CT‐based hip impingement simulation of a modified FABER test. Orthop J Sports Med. 2021;9:2325967121990629. 10.1177/2325967121990629 PMC8167016 34104657

[31] Yığman GT , Atalay HÖ , Dokuyucu EE . Ischiofemoral impingement: Morphologic associations, pelvic asymmetry, and clinical correlates in a retrospective observational study. Acad Radiol. 2025;32:7397‐7406. 10.1016/j.acra.2025.09.023 41047291

[32] Khodair SA , Ghieda UE , Elsayed AS . Ischiofemoral impingement syndrome: Spectrum of MRI findings in comparison to normal subjects. Egypt J Radiol Nucl Med. 2014;45:819‐824.

[33] Itthipanichpong T , Jaidee K , Wipaswatcharayotin P , et al. Hip external rotation decreases ischiofemoral distance: A positive correlation between cadaveric and fluoroscopic studies. Arthroscopy. 2025;41:207‐213. 10.1016/j.arthro.2024.03.038 38593926

